# Simultaneous submission of seven CTSA proposals: UM1, K12, R25, T32-predoctoral, T32-postdoctoral, and RC2: strategies, evaluation, and lessons learned

**DOI:** 10.1017/cts.2024.14

**Published:** 2024-01-25

**Authors:** Carolina Lema, Kwai Wa Cheng, Delanderia M. Anderson, Charles C. Miller, Daniel D. Karp, David D. McPherson, Satya Sree N. Kolar

**Affiliations:** 1 Center for Clinical and Translational Sciences (CCTS), The University of Texas Health Science Center at Houston, Houston, TX, USA; 2 Department of Internal Medicine, Division of Cardiology, The University of Texas Health Science Center at Houston, McGovern Medical School, Houston, TX, USA; 3 Department of Cardiothoracic and Vascular Surgery, The University of Texas Health Science Center at Houston, McGovern Medical School, Memorial Hermann Heart & Vascular Institute, Houston, TX, USA; 4 Department of Investigational Cancer Therapeutics, The University of Texas MD Anderson Cancer Center, Houston, TX, USA

**Keywords:** CTSA, PAR-21-293, CCTS, grant writing, UM1

## Abstract

Translation is the process of turning observations in the research laboratory, clinic, and community into interventions that improve people’s health. The Clinical and Translational Science Awards (CTSA) program is a National Center for Advancing Translational Sciences (NCATS) initiative to advance translational science and research. Currently, 64 “CTSA hubs” exist across the nation. Since 2006, the Houston-based Center for Clinical Translational Sciences (CCTS) has assembled a well-integrated, high-impact hub in Texas that includes six partner institutions within the state, encompassing ∼23,000 sq. miles and over 16 million residents. To achieve the NCATS goal of “more treatments for all people more quickly,” the CCTS promotes diversity and inclusion by integrating underrepresented populations into clinical studies, workforce training, and career development. In May 2023, we submitted the UM1 application and six “companion” proposals: K12, R25, T32-Predoctoral, T32-Postdoctoral, and RC2 (two applications). In October 2023, we received priority scores for the UM1 (22), K12 (25), T32-Predoctoral (20), and T32-Postdoctoral (23), which historically fall within the NCATS funding range. This report describes the grant preparation and submission approach, coupled with data from an internal survey designed to assimilate feedback from principal investigators, writers, reviewers, and administrative specialists. Herein, we share the challenges faced, the approaches developed, and the lessons learned.

## Introduction

The National Center for Advancing Translational Sciences (NCATS) defines translational science as “the field of investigation focused on understanding the scientific and operational principles underlying each step of the translational process [1]” where translation refers to “the process of turning observations in the laboratory, clinic, and community into interventions that improve the health of individuals and the public [2].” The Clinical and Translational Science Awards (CTSA) program is an NCATS initiative that supports translational science and translational research in 64 medical research institutions (CTSA hubs) across the nation at a total cost of approximately half a billion dollars, making it the largest extramural program at NIH. Such considerable investment responds to the pressing need to accelerate the translation of science into clinical practice, a process estimated to take an average of 17 years for only 14% of new discoveries [3]. In 2021, NCATS released a new Funding Opportunity Announcement (FOA), PAR-21-293, inviting applications from all existing and new CTSA hubs [1]. The new FOA focuses on promoting the development, validation, and dissemination of scientific and operational innovations that improve the efficiency and effectiveness of clinical translation and address health disparities by delivering the benefits of translational science to all [4].

In 2006, the Center for Clinical Translational Sciences (CCTS) started with two academic institutions: the University of Texas Health Science Center at Houston (now UTHealth Houston), the University of Texas M.D. Anderson Cancer Center, and two hospital systems: Memorial Hermann and Harris County Health System, laying the cornerstone for a well-integrated, high-impact CTSA hub in Texas. Presently, the CCTS has expanded to include four more academic institutions: Rice University, The University of Texas at Tyler, The University of Texas Rio Grande Valley, and Texas Tech University Health Sciences Center El Paso, along with their respective institution-affiliated hospitals. Located in Texas, each partner institution brings communities with unique geographic, demographic, cultural, and socioeconomic characteristics and challenges encompassing ∼ 23,000 sq. miles and over 16 million residents (4% of the US population) with one of the highest levels of diversity in the nation.

The primary goal of the CCTS is to fully integrate translational science into all its activities, initiatives, and projects, from training and career development to community engagement and implementation of innovative interventions in low-resourced locations. In May 2023, our hub simultaneously submitted seven applications to NIH-NCATS: UM1 (PAR-21-293), K12 (PAR-21-336), R25 (PAR-21-339), T32-Predoctoral (T32-Pre, PAR-21-337), T32-Postdoctoral (T32-Post, PAR-21-338), and two RC2 (PAR-21-340). The overall process involved experienced leadership, dedicated administrative support, intense collaboration across partner institutions, and cohesive teamwork. In October 2023, we received fundable scores for the UM1 (22), K12 (25), T32-Predoctoral (20), and T32-Postdoctoral (23). This communication describes the process of preparing a UM1 application along with six companion grant proposals. The main motivation behind this venture was to maximize time and optimize efforts during the current fourth year of funding. By sharing the challenges faced, approaches taken, and lessons learned, we anticipate this publication will provide invaluable insight to the existing and prospective CTSA hubs that plan to submit multiple CTSA applications concurrently.

## Grants, writers, and reviewers

In 2021, NCATS released the new UM1 solicitation along with five companion funding opportunities (K12, R25, T32-Pre, T32-Post, and RC2). Of the five companion applications, the required K12 Clinical Scientist Institutional Career Development Program Award application is mandatory for the UM1 review. A successfully funded seven-year UM1 application dictates the funding of all companion applications (with a five-year funding cycle), including the companion K12. Any unsuccessful application can be resubmitted following a successful UM1 application. The K12 calls for applicants to propose innovative institutional research career development programs designed to prepare late-stage postdoctoral fellows and junior faculty scholars in clinical and translational science. The NIH Research Education Program (R25), another UM1 companion, supports short-term research educational activities with a primary focus on research experiences related to clinical and translational research stages: preclinical (T1), clinical (T2), clinical implementation (T3), and public health (T4). The T32-Pre and T32-Post Research Training Grants are two independent programs focused on enhancing the research training of individuals seeking a doctoral degree (PhD) or postdoctoral experience while contributing to a heterogeneous pipeline of clinical and translational scientists. Finally, the High Impact Specialized Innovation Program (RC2) supports nonclinical trial initiatives that develop unique hub capabilities and resources to address critical gaps and/or roadblocks in clinical and translational science at an awarded UM1 CTSA hub. We submitted two RC2 applications: Program for Opioid Management Implementation for Patients in Resource-limited Settings (PROMISE) and Model for Outpatient Delivery of Excellence in Leukemia Treatment (MODEL-T). The principal investigator (PI) of each application was the lead and primary writer of the proposal. For the UM1, the CTSA contact PI designated the Element leaders who led the UM1 Elements/Modules writing task. There were two steps involved in the review process: internal and external reviews. During the internal review, the proposal drafts were exchanged between the writers (proposal PIs) who reviewed each other’s work. Next, the revised proposals were assigned to nine contributors, including members from our External Advisory Board (EAB) and PIs from other CTSAs, who were requested to serve as external reviewers.

## Administration (Admin) team

The CCTS Admin team included twelve members: seven Program Managers, two Administrative Assistants, and three Support Staff. All Program Managers worked under the CCTS Executive Director, who liaised between the PIs, writers, reviewers, and the institutional UTHealth Sponsored Projects Administration (SPA) specialists. Each Program Manager was designated to assist one PI. The Program Manager shared the writing instructions, checklists, and timelines with the PI and was responsible for document accuracy, completeness, and adherence to the submission timeline. The scientific documents included the research plan, specific aims page, project narrative, and abstract. The nonscientific documents included the biosketches, letters of intent, letters of support, data management/sharing plans, and other accessory documents as required by the FOA. The Program Managers were also delegated to work on (i) formatting tables, figures, and references; (ii) working with the CCTS Financial Director on the budget; and (iii) assisting the CCTS Executive Director in planning and scheduling, communicating with PIs, and setting deadlines. The Admin Assistants and Support Staff were tasked with scheduling and/or facilitating virtual and/or in-person meetings, printing materials, and providing logistical support during the two-day writing retreat and in-person final review.

## Partner institutions

The leaders from our hub’s six partner institutions were critically involved in the proposal development process by becoming actively engaged in proposal writing and/or internal review. At the EAB meeting, the institutional leaders shared “Progress-to-Date” and “Going-Forward” activities, outlining their ongoing and prospective initiatives. Additionally, their insight into other events, including our Grant Writing Retreat, led to the successful integration of the six Key Components: Community and Stakeholder Engagement (C&SE), Dissemination and Implementation (D&I), Diversity, Equity, Inclusion, and Accessibility (DEI-A), Workforce Development (WD), Health Informatics (HI), and Evaluation (EV) into all proposals.

## Approach

**Figure 1.**
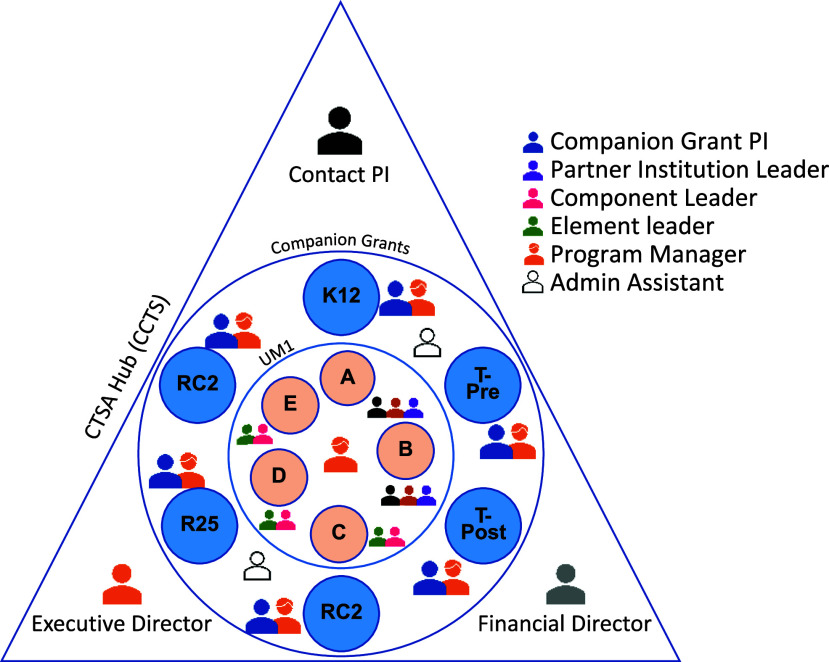
Organization of the CCTS (CTSA hub) team members involved in the simultaneous submission of a UM1 along with 6 companion grants. The CTSA contact PI, Executive Director, and Financial Director planned and directed the development and submission processes. For each grant, a Program Manager was designated to exclusively assist the companion grant PI who served as the team leader. For the UM1, the CTSA contact PI designated the Element leaders who worked closely with the Components (Elements C, D, and E) and/or partner institution leaders (Elements A and B).

The new 2021 FOA consisted of separate UM1, K12, T32-Pre, T32-Post, R25, and RC2 applications and required programmatic structural and functional reorganization of the UM1. To accomplish a single “voice” when many writing teams were in play, we: (i) built writing teams using previously established working groups; (ii) set deadlines that were put out by the CTSA contact PI, followed by specific instructions from the CCTS executive director; and (iii) defined roles and responsibilities for all team members. The simultaneous submission of seven proposals culminated in logistical advantages, including: (i) the creation of supporting document templates that were “tailored” to each proposal; (ii) the effective and time-saving delivery of instructions and communications during meetings or via e-mail; and (iii) the successful tracking of all activities by a master timeline. The magnitude of our approach was highlighted when we estimated ∼ 38,616 hours (∼4.4 years!) time was devoted by all participants toward this ambitious project. Using the standard 2,000 hours per year for a full-time employee, the total hours are equivalent to 19 person-years. A diagram of the hub team members involved in this endeavor is shown in Figure 1.

## Goals

To address the new FOA, we identified three key goals: (i) To successfully transition from a 16 Components organizational structure to five operational units defined as Elements (A–E) and their subunits or Modules; (ii) To integrate our D&I capabilities into all initiatives which required us to redefine D&I as a Key Component; and (iii) To create a strong sense of community and teamwork within our hub where the PIs, writers, reviewers, Admin team, and SPA specialists felt motivated to actively contribute to a joint effort with a focus on collaborative teamwork, leading to the simultaneous submission of seven CTSA proposals. The specific approach used to accomplish these three key goals is shown in Table 1.

First, to transition from Components to Elements, the UM1 FOA was “dissected” to match the existing 16 Components with the newly proposed Elements A-E and their Modules and to build working groups with designated leaders (Table 2). The Elements/Modules leaders were designated by the CTSA contact PI with approval from the existing Component Directors. The working groups received several supporting materials from checklists to specific writing instructions and general formatting guidelines. Similar materials were created for the companion K12, T32-Pre, T32-Post, R25, and RC2 proposals (Table 3). All supporting materials were developed by the CCTS Admin team for the benefit of PIs, writers, and reviewers in an effort to facilitate consistency during writing and review.

Second, prior to this submission, D&I was a part of our C&SE Component. To promote its new centralized role, the existing D&I leader met with the Component Directors to provide individual feedback and promote the integration of D&I. The new FOA initiated us to redefine D&I as an individual Key Component along with DEI-A, C&SE, WD, HI, and EV, which were weaved into all seven grants. This strategy was further sustained by having the D&I, C&SE and HI leaders as MPIs of the UM1 proposal. To promote a collaborative effort and disseminate individual Key Component Directors input, we conducted a two-day in-person writing retreat. The retreat brought together the leaders of all partner institutions, components, and companion grants to discuss potential opportunities to enrich and integrate the six identified key components into current and future efforts.

Finally, the writing retreat also facilitated accomplishing our third goal: to approach the seven-proposal submission as a team effort. Additionally, our EAB meeting, held seven months prior to submission, created a perfect opportunity to provide the time and space needed for autoevaluation, redirection, and integration as a cohesive team. Transitioning from “less siloed to more bonded” was our EAB’s recommendation, which we adapted as the motto going forward. Pursuing the simultaneous submission of seven proposals motivated the leaders of each partner institution, Component, and companion grant to synthesize innovative ways to integrate all our programs and work as a team from start to finish.

**Table 1.** Goals, challenges, and approaches

Goal	Challenge	Existing state	Approach / Activity
1. Transition from Components (16) to Elements (5)	FOA was restructured for first time since its inception (2006)	Currently associated Components (e.g., RKS and BERD; CS&E and ISP; BERD and HI; CRUs and HI) Existent TIN (precursor of HLT)	Dissected FOA Matched existing Components to Elements Assigned a writing team for each element and appointed an element leader to guide the writing team
2. Weave D&I, DEI-A, C&SE, WD, HI, and EV into all Elements	D&I tools and approaches were new for most of the partner institutions, Components, and companion grants	Some Components functionally integrated (e.g., D&I and C&SE; DEI-A and ISP)	Grant Writing Retreat: Identified six Key Components Partner institutions, Component, and companion grant writers submitted a draft reflecting the integration of Key Components
3. Create a common goal/opportunity: the CTSA grant renewal and facilitate collaboration among all partner institutions, Components, and companion grants	Large group: six institutions across Texas and CCTS organized in 16 Components Multidisciplinary and diverse team: each component is led by an established independent investigator with diverse background and unique work style and schedule	Close coordination between leadership and CCTS Admin team Established logistics for meetings and announcements EAB meeting conducted annually	Multiple in-person meetings including partner institutions, grant PIs, Component directors, and Admin team (e.g., EAB meeting, writing retreat, in-person reviews) Every partner institution, Component, and companion grant submitted a draft addressing three questions: what we have done (accomplishments) what we are planning to do (renewal aims) how we can collaborate within our hub

BERD = Biostatistics, Epidemiology, and Research Design; C&SE = Community and Stakeholder Engagement; CRUs = Clinical Research Units; D&I = Dissemination and Implementation; DEI-A = Diversity, Equity, Inclusion, and Accessibility; EV = Evaluation; HI = Health Informatics; HLT = Hub Liaison Team; TIN = Trial Innovation Network; ISP = Integrating Special Populations; RKS = Regulatory Knowledge & Support; WD = Workforce Development.

**Table 2.** Writing teams

Component	Element/Module	Leader
Administration	A	Element A has no leader (FOA). The CTSA contact PI led the writing activities
C&SE, HI, WD, EV, DEI-A, D&I, Administration	B	CTSA contact PI
WD and Team Science	C1	C&SE Component Director
C&SE and ISP	C2	
D&I	C3	
CRUs, BERD, RKS	D1	TIN Component Director
Pilot Projects	D2	
HI	D3	
LHC	E	LHC Component Director

BERD = Biostatistics, Epidemiology and Research Design; C&SE = Community and Stakeholder Engagement; CRUs = Clinical Research Units; D&I = Dissemination and Implementation; DEI-A = Diversity, Equity, Inclusion and Accessibility; EV = Evaluation; HI = Health Informatics; LHC = Learning Healthcare; TIN = Trial Innovation Network; ISP = Integrating Special Populations; RKS = Regulatory Knowledge & Support; WD = Workforce Development.

**Table 3.** Supporting materials (proposal)

Material	Content
Element and Component Specific Aims (UM1)	Element description Page limit Element leader Component Specific Aims
Key Components, Components & Companion Grants (UM1)	Components and companion grants with their respective leaders
Writing instructions adapted from FOA (UM1)	Element/module description Matching components Element leaders Writing team members Formatting info (font, size, spacing, margins)
Writing instructions adapted from FOA (K12, R25, T32-Pre, T32-Post, and RC2s)	Specifically tailored for each grant. Includes: Key terms/description Scope and purpose Expected content Formatting info (font, size, spacing, margins)
Additional documents checklist (UM1, K12, R25, T32-Pre, T32-Post, and RC2s)	Specifically tailored for each grant includes: Budget guidelines Budget justification instructions Required scientific documents and nonscientific documents: Each with a short description, page limit, and due date
Biosketches (UM1, K12, R25, T32-Pre, T32-Post, and RC2s)	Instructions Updated NIH template Samples
Tables, Figures, Vignettes, Abbreviations boxes (UM1, K12, R25, T32-Pre, T32-Post, and RC2s)	Templates Figure and Vignettes suggestions List of abbreviations
References (UM1, K12, R25, T32-Pre, T32-Post, and RC2s)	Instructions for bibliography and citation style EndNote libraries guideline
CTSA Grant Writing Survey	Five roles: PI, writer, reviewer, Admin team, and SPA specialist Five-to-ten minutes, ∼20 questions/role Designed using Qualtrics

## Submission timeline & key meetings

**Figure 2.**
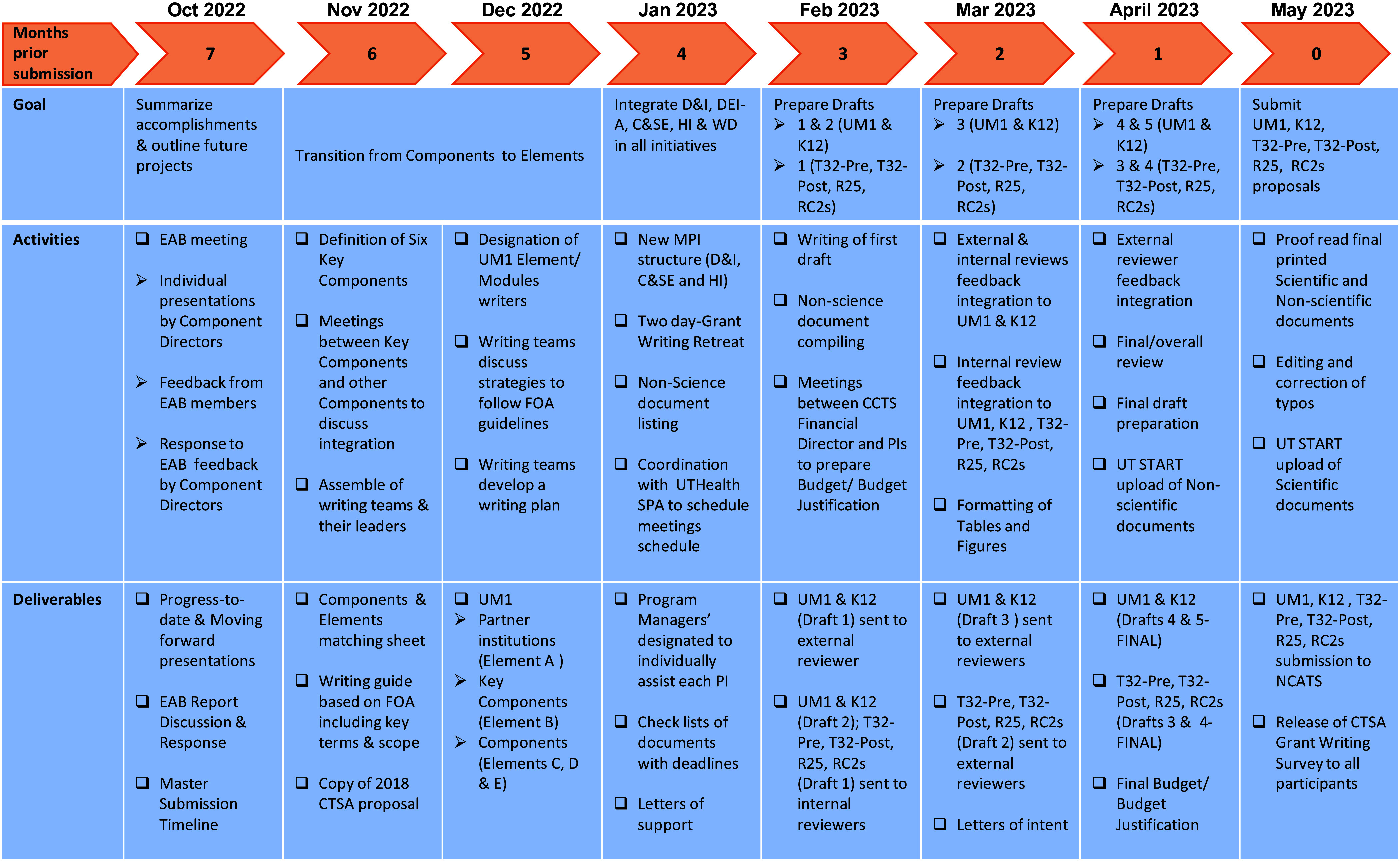
Submission timeline. Simplified version of the master timeline used for the simultaneous submission of a UM1 along with 6 companion proposals.

The entire grant writing effort was coordinated using a master timeline developed by the Admin team seven months before submission. Figure 2 shows a summarized version of the master submission timeline. The original document was designed using a calendar format. All activities included tasks that were planned on a bi-weekly basis. The tasks were classified as: (i) proposal writing, (ii) draft review, (iii) preparation of required nonscientific documents, and (iv) proposal submission. Preceding the start of each task, the CTSA contact PI and the CCTS Executive Director met to discuss and elaborate on a detailed strategic plan, including specific subtasks and associated deadlines. This was shared with all hub members via a presentation during our weekly mandatory Monday CCTS meeting. This hybrid meeting allowed for the active engagement of all hub members, including PIs, members from our partner institutions in Northeast, South, and West Texas, Component Directors, and the Admin team. Occasional absentees were provided with a video recording. Seven months pre-submission, the well-established CCTS weekly meeting became pivotal as it served as an effective channel for open discussion, feedback, updates, and logistical communication.

Additionally, the Admin team met virtually with the CCTS Executive Director every two weeks for four months, weekly for three months, and daily for two weeks pre-submission. The Admin meetings were used to share regular updates and obtain feedback to resolve emerging problems and discuss strategies to provide PIs with the best support. This meeting was held in person the week before submission to discuss grant document receipt and grant submission status. The CTSA contact PI met in person with the CCTS Executive Director two times a week during the last four months and weekly with the financial director for two months pre-submission.

During the whole process, the Admin team was committed to answering any questions and providing prompt support. Further, the CCTS Executive Director was available around the clock to meet with PIs, Admin team members, and the institutional SPA specialists with whom regular communication was established for guidance, input, and feedback related to proposal submission.

## Evaluation

To collect feedback and improve the future submission process, following the seven-grant submission endeavor, the CCTS Admin and Evaluation teams created an anonymous post-submission CTSA grant writing survey (Qualtrics software, Provo, UT). Participants were asked to select among five defined roles: PI, writer, reviewer, Admin team, and SPA specialist. The five-to-ten-minute survey included about 20 questions/role and was distributed via e-mail (hyperlink and QR code) by the CCTS Executive Director to all 56 participants involved in grant writing, review, and/or submission processes.

## Key survey results

- Thirty-four out of fifty-six participants completed the online survey (response rate of 61%). Fifteen percent of the respondents identified themselves as PIs, 32% as writers, 23% as reviewers, and 30% as administrators (Admin team and SPA specialists). The distribution of the survey respondents by grant demonstrated that 22% worked on UM1, 12% on K12, 13% on T32-Pre, 14% on T32-Post, 15% on R25, 12% on RC2-PROMISE, and 11% on RC2-MODEL-T, showing an even distribution across all grants.
- All participants were asked to share three words that reflected their grant preparation experience. The words provided by the survey participants were pooled by role, and the percentage of words with a positive connotation was obtained. The data reflected an overall positive experience in 55% of PIs, 58% of writers, 76% of reviewers, and 47% of administrators.
- Forty-seven percent of the respondents indicated involvement in the previous grant renewal in 2018, indicating that over half (53%) of the participants were new to the current proposal development process.
- When asked for feedback, the survey respondents identified 4 ± 1 as the appropriate number of drafts for all proposals. This was in agreement with the UM1 and K12 writers delivering five drafts: initial, post-solo-external, post-internal, post-external, and final (indicated as drafts 1–5 in Figure 2); and the T32-Pre, T32-Post, R25, and RC2 proposals having four drafts: initial, post-internal, post-external, and final (indicated as drafts 1–4 in Figure 2).
- The time allocated to address the reviewer’s comments ranged from 1 week (fourth and fifth drafts) to 2 weeks (first to third draft). Eighty-four percent of the combined group of writers and reviewers agreed that the time between drafts was appropriate.
- For preferred communication practices, 38% of the writers chose virtual meetings, 32% selected e-mail, 16% chose in-person meetings, and 14% selected phone.
- The writers chose the biosketch template and samples (87%), checklists with deadlines (87%), and the submission timeline (73%) as the top-three most helpful support materials for nonscientific documents.
- As for the scientific document’s supporting materials, 79% of the writers found the reviewer’s feedback most beneficial, followed by the follow-up emails with deadlines (73%), the writing instructions, templates and links (67%), and the use of Google Drive as a draft repository and tracking (50%).
- The final review of all proposals was conducted in person, where printed documents were used to receive edits and comments. Forty percent of the reviewers endorsed this approach as effective, and 60% considered it somewhat effective.
- Seventy-four percent of the PIs, 77% of the writers, and 100% of the reviewers reported that the overall Admin support was very effective.
- Fifty percent of the PIs reported being very satisfied regarding their overall experience, while 33% were satisfied, and 17% were neither dissatisfied nor satisfied.

## Lessons learned & recommendations

- **Early start.** Although our EAB meeting marked the formal beginning of the seven-proposal development process (seven months pre-submission), most planning meetings were initiated about ten months before submission. All survey respondent PIs agreed that the starting time was appropriate.
- **Pre-established working groups.** Most of the PIs have been working together since the inception of the hub in 2006. This well-established existing infrastructure provided the foundation required for the simultaneous preparation and submission of seven CTSA proposals.
- **Weekly CCTS and Admin meetings.** The weekly CCTS and Admin meetings served as the reference point to “keep moving forward.” The existent weekly CCTS meeting was used as regular assembly time to receive weekly updates from all leaders including those from our partner institutions. Steered by the hub contact PI and attended by all seven grant PIs, writers, and the Admin team, its goal was to provide strategic direction by sharing plans and establishing deadlines. The Admin meeting was led by the CCTS executive director to discuss logistics and execute scheduled tasks in a timely manner via coordination with the seven Program Managers who were working side-by-side with PIs, writers, and internal reviewers. Both meetings served as an excellent opportunity to keep the teams motivated. Acknowledging the effort of each team member and providing the necessary support was critical to maintaining an upbeat morale, constant engagement, and high productivity during a lengthy, draining, and often frustrating process. Additionally, these meetings allowed for open group discussions, with significant advantages over one-on-one meetings, as they saved time and the whole group benefited from the shared information and knowledge.
- **Early engagement of the institutional Grants Administration office (SPA).** Engaging with SPA specialists seven months pre-submission to inform them about the multiple proposal submission and build a SPA-inclusive timeline was critical to establish a collaborative-cohesive team. This effort was paramount to dealing with a 48-hour submission delay caused by a system glitch on our institutional submission portal. Additionally, having an internal deadline twelve business days prior to the application due date allowed for time to fix the technical glitch and still submit the applications before the NCATS application due date.
- **New budget guidelines.** For the UM1, PAR-21-293 requires the budget to be organized by elements/modules. Discussing the new budget structure and thus ensuring that the PIs are fully aware of the change early in the process will save time and effort. From our experience, we learned that facilitating meetings between the Element leaders and the CCTS Financial Director well before beginning the writing process is crucial. Importantly, securing institutional funding could be a significant challenge, especially for new applicants. Therefore, addressing these conversations earlier will allow for a time-efficient budget preparation process.
- **Individual point-of-contact Admins.** A designated Program Manager working with each element leader/PI aided the Admin team to execute assigned tasks simultaneously while tracking proposal progression. Our survey demonstrated that 50% of the respondent PIs approved this approach.
- **One Admin accountable for a few tasks**. Designating one Program Manager to a specific proposal allowed for seamless proposal development progress until submission. As previously eluted, the Admin meetings evaluated progress and addressed difficulties. Each Admin team member was accountable for the tasks assigned. Our experience provides validation that only one Admin member should upload documents onto the institutional submission portal. This process allows for consistency with file names, avoids wrong versions and/or duplications of uploaded documents, and provides a single point-of-contact for the contact PIs and SPA specialists. Ideally, a second Admin member who is well aware of all proceedings should be designated as a backup.
- **Internal review.** All Component Directors of the UM1 and PIs of the companion proposals were invited to serve as internal reviewers. However, this approach resulted in a variable number of reviewers per draft and led to coordination challenges owing to varying schedules around a single deadline. Based on this experience, we recommend limiting one to two internal reviewers per draft.
- **Internal submission deadline.** As mentioned earlier, having twelve extra business days makes a difference between a chaotic and a less chaotic submission process, especially during a multiple-proposal submission endeavor. In our case, the twelve-day internal deadline allowed for enough time to maneuver a last-minute technical glitch while still keeping the submission prior to the application due date.
- **Survey.** To avoid overwhelming the survey respondents, questions in our survey did not require reasoning behind the respondent’s choice. Nonetheless, this hampered our ability to capture valuable information for further improvement.

## Conclusion

A seven-grant proposal development for simultaneous submission was an enormous team effort that required high-level strategic leadership, planning, dedication, organization, and effective communication. Receiving fundable scores for the UM1 (22), K12 (25), T32-Pre (20), and T32-Post (23) was very gratifying for the team as a whole and added a great deal of energy and motivation for the resubmission of our R25 and RC2 proposals.
